# Editorial: Traditional Chinese medicine for depression and anxiety

**DOI:** 10.3389/fpsyt.2023.1217886

**Published:** 2023-07-12

**Authors:** Xinjing Yang, Chuan Shi, Tuya Bao, Zhangjin Zhang

**Affiliations:** ^1^Department of Traditional Chinese Medicine, South China Hospital of Shenzhen University, Shenzhen, China; ^2^Psychological Assessment Center, Peking University Sixth Hospital, Beijing, China; ^3^School of Acupuncture-Moxibustion and Tuina, Beijing University of Chinese Medicine, Beijing, China; ^4^School of Chinese Medicine, Li Ka Shing Faculty of Medicine, The University of Hong Kong, Hong Kong, Hong Kong SAR, China

**Keywords:** Traditional Chinese Medicine, depression, anxiety, acupuncture, Chinese herbal medicine, treatment

Depression and anxiety are the two most common mental disorders, resulting in enormous misery and loss of health (1). The high heterogeneity of clinical symptoms and etiologies of depression and anxiety results in low response and remission rates of current single-target antidepressants and anxiolytics (2, 3). Traditional Chinese Medicine (TCM) has been used in treating depression and anxiety for many years. Compared to conventional medicine, “holism” and “treatment based on syndrome pattern differentiation” are essential characteristics and advantages of TCM. TCM holism refers to cognition of the wholeness of the human body and the relationship between humans, nature, and society. Treatment based on syndrome pattern differentiation means that after syndrome pattern differentiation (inspection, listening and smelling, inquiry, and palpation), TCM doctors determine the treatment principles and choose and carry out concrete therapies based on patients' conditions. TCM has a multi-target and extensive regulation in treating depression and anxiety and is considered an alternative or adjunct therapy for depression and anxiety with increasing evidence of effectiveness (4, 5).

However, TCM's clinical effectiveness and underlying mechanisms for depression and anxiety are not fully illustrated. Accordingly, we initiated this Research Topic to provide a forum for state-of-the-art research on TCM for depression and anxiety. Here, we highlight relevant results and conclusions from the five articles published on the Research Topic.

## 1. Chinese herbal medicine and formula for depression

Sun et al. comprehensively reviewed literature focused on the antidepressant activities of herbal medicine and identified 45 antidepressant herbal medicines from various studies *in vitro* and *in vivo*. The antidepressant mechanisms may involve multiple signaling pathways regulating neurotransmitters, neurogenesis, anti-inflammation, antioxidation, endocrine, and microbiota. Significantly, herbal medicines can modulate a broader spectrum of cellular pathways and processes to relieve depression and avoid the side effects of antidepressant drugs. This review provides molecular insights into novel antidepressant drugs for Chinese herbal medicine in the form of monomers and extracts.

In clinical practice, TCM doctors usually combine several herbs known as formulas to achieve a better therapeutic effect. Xiaoyao powder, Yueju pills, and Ganmai Dazao decoction are classic TCM formulas for treating mental disorders.

Wang H. et al. conducted a network pharmacology and molecular docking study to explore the mechanism of Sinisan Formula (SNSF) in depression. SNSF is a well-known TCM formula consisting of four herbs: Bupleurum chinense (Chaihu), Paeoniae Radix Alba (Shaoyao), Aurantii Fructus Immaturus (Zhishi), and licorice (Gancao). In SNSF, 91 active compounds connect to 112 depression-related targets. SNSF may exert its antidepressant effects by regulating the signaling pathway of 5-hydroxytryptamine, dopamine, gamma-aminobutyric acid, and neuroactive ligand-receptor interactions. Research on TCM formulas is challenging. The interactions of various components of different herbals complicate the action mechanisms. The methodology and process of this study provide a reference for other studies on TCM formulas.

## 2. Acupuncture for anxiety and depression

Acupuncture is the most widely used traditional and complementary medicine, used in 113 of 120 countries predominantly for pain relief and in various other conditions (6, 7). Acupuncture has a moderate or significant effect with moderate or high certainty evidence in eight diseases or conditions, such as post-stroke aphasia, pain, and allergic rhinitis (8, 9). Although research on acupuncture for mental disorders is increasing and has shown positive effects in recent years, more evidence of acupuncture for mental disorders is needed (10).

There are many acupuncture-related therapies, such as acupuncture, electroacupuncture, warm acupuncture, and moxibustion. In this collection, Wang X. et al. found that acupuncture-related therapies can alleviate anxiety and depression among irritable bowel syndrome with diarrhea (IBS-D) patients with a higher safety level by using network meta-analysis. In addition, combining acupuncture-related therapies with other therapies has a high overall benefit and safety. However, the sample sizes of the clinical trials are relatively small, and the acupoints selected in each study are quite different, making it challenging to guide clinical practice. More high-quality clinical trials can help narrow the gap between research and clinical usage.

As a non-pharmaceutical therapy, acupuncture is of high safety, especially for patients in a particular period. For example, acupuncture is efficacious for moderate-to-severe nausea and vomiting during pregnancy, and the combination of acupuncture and doxylamine-pyridoxine may achieve better effectiveness than each treatment alone (11). In this collection, Hou et al. designed a clinical trial to investigate the effects of electroacupuncture on perioperative anxiety and stress responses in patients undergoing surgery for gastric or colorectal cancer. As a pilot trial, this study was designed to conduct in one single center with a relatively small sample size. We expect professional organizations, societies, and acupuncture trialists to develop international guidance facilitating the development of acupuncture treatment protocol (12).

## 3. Other TCM therapies for anxiety and depression

TCM emphasizes that excessive emotions cause disease, and regulating emotions can cure disease. Li et al. found that conventional treatment combined with 4–12 weeks of mindfulness treatment can significantly improve the anxiety and depression symptoms of patients with insomnia. TCM mind-body exercise therapies, such as Tai Chi and Baduanjin, also positively affect anxiety and depression (13).

## 4. Conclusions and future directions

This collection of papers provides the latest information and insights on TCM for treating depression and anxiety. TCM emphasizes a holistic view and individualized treatment. The research methodology of modern medicine is only partially fit for TCM. We need to explore a new research methodology in line with the characteristics of TCM. Modern science is tackling new approaches focused on individualization to handle the clinical heterogeneity and comorbidity inherent in mental disorders. For example, a study discovered biomarkers for diagnosis-shared symptoms among mental disorders by using a personalized fMRI approach, leading to personalized interventions (14). The study may shed light on how to conduct research about TCM in terms of individualization.

In conclusion, a more reasonable clinical trial design and the application of modern science and technology will help us better elucidate the clinical efficacy and mechanism of TCM in treating depression and anxiety.

## Author contributions

XY wrote the first draft of the manuscript. CS, TB, and ZZ contributed to manuscript revision, read and approved the submitted version. All authors contributed to the article and approved the submitted version.

## References

[1] WHO. Launch of the WHO World Mental Health Report: Transforming Mental Health for All Department of Mental Health and Substance Use. (2022). Available online at: https://www.who.int/director-general/speeches/detail/launch-of-the-who-world-mental-health-report–transforming-mental-health-for-all-department-of-mental-health-and-substance-use (accessed June 17, 2022).

[2] Hasler G. Pathophysiology of depression: do we have any solid evidence of interest to clinicians?. World Psychiatr. (2010) 9:155–61. 10.1002/j.2051-5545.2010.tb00298.x 20975857 PMC2950973

[3] Thibaut F. Anxiety disorders: a review of current literature. Dialogues Clin Neurosci. (2017) 19:87–8. 10.31887/DCNS.2017.19.2/fthibaut 28867933 PMC5573565

[4] Amorim D Amado J Brito I Fiuza SM Amorim N Costeira C. Acupuncture and electroacupuncture for anxiety disorders: a systematic review of the clinical research. Complement Ther Clin Pract. (2018) 31:31–7. 10.1016/j.ctcp.2018.01.008 29705474

[5] Li C Huang J Cheng YC Zhang YW. Traditional Chinese Medicine in Depression Treatment: From Molecules to Systems. Front Pharmacol. (2020) 11, 586. 10.3389/fphar.2020.00586 32457610 PMC7221138

[6] WHO. WHO Traditional Medicine Strategy: 2014-2023. Geneva: WHO (2013).

[7] WHO. WHO Global Report on Traditional and Complementary Medicine. Geneva: WHO (2019).

[8] Lu L Zhang Y Ge S Wen H Tang X Zeng JC. Evidence mapping and overview of systematic reviews of the effects of acupuncture therapies. BMJ Open. (2022) 12:e056803. 10.1136/bmjopen-2021-056803 35667716 PMC9171228

[9] Lu L Zhang Y Tang X Ge S Wen H Zeng J. Evidence on acupuncture therapies is underused in clinical practice and health policy. BMJ. (2022) 376:e067475. 10.1136/bmj-2021-067475 35217525 PMC8868048

[10] Armour M Smith CA Wang LQ Naidoo D Yang GY MacPherson H. Acupuncture for depression: a systematic review and meta-analysis. J Clin Med. (2019) 8:1140. 10.3390/jcm8081140 31370200 PMC6722678

[11] Wu XK Gao JS Ma HL Wang Y Zhang B Liu ZL. Acupuncture and doxylamine-pyridoxine for nausea and vomiting in pregnancy: a randomized, controlled, 2 × 2 factorial trial. Ann Intern Med. (2023) 10, 7326./M22-2974. 10.7326/M22-2974 37335994

[12] Fei YT Cao HJ Xia RY Chai QY Liang CH Feng YT. Methodological challenges in design and conduct of randomised controlled trials in acupuncture. BMJ. (2022) 376, e064345. 10.1136/bmj-2021-064345 35217507 PMC8868049

[13] Abbott R Lavretsky H. Tai Chi and Qigong for the treatment and prevention of mental disorders. Psychiatr Clin North Am. (2013) 36:109–19. 10.1016/j.psc.2013.01.011 23538081 PMC3917559

[14] Li M Dahmani L Hubbard CS Hu Y Wang M Wang D. Individualized functional connectome identified generalizable biomarkers for psychiatric symptoms in transdiagnostic patients. Neuropsychopharmacology. (2023) 48:633–41. 10.1038/s41386-022-01500-4 36402836 PMC9938230

